# Effectiveness and mechanism of moxibustion in treating chronic non-specific low back pain: study protocol for a multicenter randomized controlled trial

**DOI:** 10.3389/fmed.2025.1664326

**Published:** 2025-09-12

**Authors:** Xuewei Wang, Caifeng Zhu, Hongping Pan, Dongsheng Liang, Nana Zhao, Mingming Wang, Bingyuan Zhou, Han Xiang

**Affiliations:** ^1^The First Clinical Medical School, Anhui University of Chinese Medicine, Hefei, Anhui, China; ^2^Third Department of Geriatrics, The Second Affiliated Hospital of Anhui University of Chinese Medicine, Hefei, Anhui, China; ^3^Fifth Department of Encephalopathy, The Second Affiliated Hospital of Anhui University of Chinese Medicine, Hefei, Anhui, China; ^4^Second Department of Acupuncture, Chuzhou Hospital of Integrated Traditional Chinese and Western Medicine, Chuzhou, Anhui, China; ^5^Department of Acupuncture, Luohu District Hospital of Traditional Chinese Medicine, Shenzhen, Guangdong, China

**Keywords:** low back pain, moxibustion, sham moxibustion, protocol, functional near-infrared spectroscopy, randomized controlled trial

## Abstract

**Introduction:**

Chronic non-specific low back pain (CNLBP) represents the most commonly encountered subtype of low back pain (LBP) in clinical practice. It has no clearly identified etiological factors and is prone to recurrence, which severely compromises patients’ quality of life. Moxibustion therapy is commonly utilized in China for managing chronic pain conditions and has demonstrated favorable clinical outcomes. However, high-quality randomized controlled trials remain scarce, and the mechanism of action of moxibustion remains unclear. This severely restricts the credibility of moxibustion therapy and its global promotion and application. Consequently, the present research aims to conduct a comprehensive evaluation of the therapeutic efficacy of moxibustion for the management of CNLBP. Additionally, this study will employ modern scientific techniques to conduct a preliminary investigation into the mechanism of action of moxibustion.

**Methods and analysis:**

This study will be conducted simultaneously across three tertiary hospitals in China. 150 participants diagnosed with CNLBP will be recruited for this study. Subsequently, these participants will be randomly assigned, following a 1:1 allocation ratio, to undergo either moxibustion or sham moxibustion intervention in accordance with the established research protocol. Treatment will be administered at an identical set of acupoints for all participants: bilateral BL23 (Shenshu), GV3 (Yaoyangguan), and GV8 (Jinsuo). Each session will last 30 min, administered three times weekly for 8 weeks, and an 8-week follow-up will be conducted after the completion of the moxibustion intervention. Change in Numerical Rating Scale (NRS) scores from baseline to the 8-week post-intervention assessment constitutes the primary outcome measure. Secondary outcomes will include assessments via the Oswestry Disability Index (ODI), Fear-Avoidance Beliefs Questionnaire (FABQ), 36-Item Short Form Health Survey (SF-36), Global Perceived Effect (GPE), and functional near-infrared spectroscopy (fNIRS). Evaluations for this research will be conducted at baseline, following the intervention (the fourth week), after the completion of intervention (the eighth week), and during the follow-up period (week 16).

**Discussion:**

The results obtained from this research are expected to indicate that moxibustion therapy can function as a highly efficacious treatment approach for managing CNLBP. Additionally, this trial will employ fNIRS technology to investigate the activation characteristics of pain-related cortical regions in the brains of CNLBP patients before and after moxibustion treatment. This will contribute to elucidating the underlying mechanisms of moxibustion.

## 1 Introduction

Low back pain (LBP), a highly common clinical condition, adversely affects patients’ work performance and social activities, imposing a substantial socioeconomic burden (1). Epidemiological research consistently shows that nearly 80% of adults have at least one LBP episode in their lifetime (2). Additionally, 22%–65% of patients report recurrence at least once a year (3). Chronic non-specific low back pain (CNLBP) is the most common subtype, accounting for 80%–90% of all LBP cases (4). It manifests as pain or discomfort between the costal margin and gluteal fold, lasting over 12 weeks with no clearly identifiable cause.

The pathogenesis of CNLBP is highly complex and has not been fully elucidated. Recent research indicates that it is associated with a dynamic interplay among biological, psychological, and social factors (5). Pharmacological interventions are no longer the primary therapeutic choice for CNLBP management due to their limited efficacy, unpredictable adverse effects, and potential risk of dependency (6, 7). Invasive therapies including epidural glucocorticoid injections and surgical procedures entail high costs and may confer greater risks of adverse events compared to non-invasive alternatives, without guaranteed resolution of patient suffering, resulting in their rare clinical adoption (8, 9). At present, clinical guidelines indicate that non-pharmacological interventions should be the first choice for effective management of chronic LBP (10). However, conservative approaches such as motor control exercises and cognitive functional therapy generally demonstrate only small effect sizes (11, 12). Consequently, it remains imperative to explore novel complementary therapies to address these clinical challenges (13). Moxibustion, as a classical traditional Chinese medicine (TCM) modality widely employed for chronic pain management, shows promise as an effective strategy for managing CNLBP.

Moxibustion is frequently combined with acupuncture in clinical practice. Moreover, it can effectively address the issue that some patients fear pain caused by invasive acupuncture, thereby reducing their anxiety toward treatment. In recent years, moxibustion has been widely adopted in China’s healthcare system. According to previous studies, its therapeutic effects primarily derive from two mechanisms: the physiological responses to heat stimulation generated through combustion of moxa (14, 15), and the chemical stimulation from pharmacological components within the herb (16). This perspective characterizes the TCM paradigm of health and disease, highlighting the crucial role of endogenous regulatory processes in maintaining homeostasis amidst external perturbations. In China, moxibustion has been extensively applied for managing various pain-related disorders including arthritis (17), dysmenorrhea (18), postherpetic neuralgia (19) and musculoskeletal pain syndromes (20), with documented beneficial clinical outcomes. Based on these experimental results, after moxibustion intervention, the patients’ pain symptoms were alleviated, demonstrating the potential clinical application of moxibustion. Furthermore, clinical observations and self-reported outcomes suggest that moxibustion may improve both physiological and psychological wellbeing, which aligns with its widespread adoption as a preventive healthcare modality in Chinese populations. Nevertheless, high-quality clinical research investigating moxibustion for CNLBP remains scarce, and its therapeutic efficacy and safety profile require further validation through rigorously designed studies.

## 2 Methods

### 2.1 Trial design

This is a multicenter, single - blind, randomized controlled trial in which 150 eligible participants will undergo 1:1 randomization to receive either verum moxibustion therapy or a sham control procedure. This study will span a total duration of 16 weeks, comprising an 8-week therapeutic intervention phase followed by an 8-week follow-up period.

**FIGURE 1:**
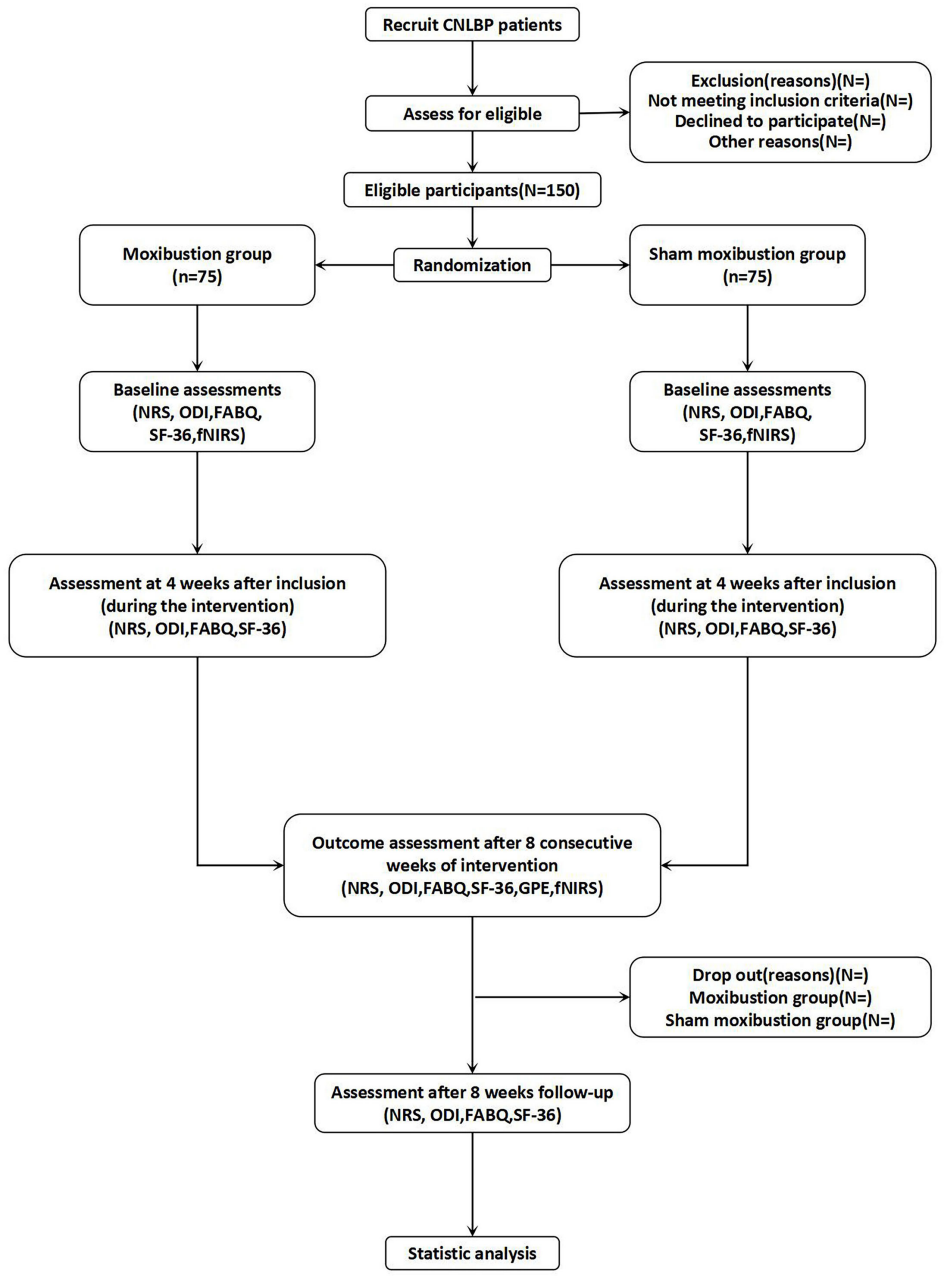
Trial flow diagram.

This trial has been registered on the International Traditional Medicine Clinical Trial Registry (ITMCTR2025001296). This study protocol adheres to the SPIRIT reporting guidelines (21). The SPIRIT checklist of this trial is given in Supplementary material. The flow diagram of this trial is illustrated in Figure 1, while the comprehensive schedule for the study is outlined in Table 1.

**TABLE 1:** Trial schedule.

	Study period			
	Inclusion	Treatment		Follow-up
Assessment	Baseline	Second	Third	Fourth
Measure point (after inclusion)	0 week	4 weeks	8 weeks	16 weeks
Inclusion criteria	✓			
Exclusion criteria	✓			
Informed consent	✓			
Randomization and allocation	✓			
Intervention		1–8 week		
NRS	✓	✓	✓	✓
ODI	✓	✓	✓	✓
FABQ	✓	✓	✓	✓
SF-36	✓	✓	✓	✓
GPE			✓	
fNIRS	✓		✓	
Adverse events	Any time during the intervention and follow-up			

✓, required; NRS, Numerical Rating Scale; ODI, Oswestry Disability Index; FABQ, Fear Avoidance Beliefs Questionnaire; SF-36, 36-Item Short Form Health Survey; GPE, Global Perceived Effect.

### 2.2 Recruitment

This study will be conducted simultaneously across three tertiary hospitals in China. Patient recruitment will be implemented through a multimodal strategy incorporating social media platforms (WeChat), local newspaper advertisements, and posters displayed in community service centers to ensure adequate participant enrollment.

### 2.3 Eligibility criteria

#### 2.3.1 Inclusion criteria

(1) Meet the diagnostic criteria for CNLBP (4);

(2) Aged between 20 and 55 years (inclusive);

(3) Score ≥ 3 on the NRS;

(4) Sign the informed consent form formulated specifically for this study.

#### 2.3.2 Exclusion criteria

(1) Misdiagnosis;

(2) LBP associated with radiculopathy or nerve root injury;

(3) History of spondylolisthesis or lumbar spine surgery;

(4) LBP resulting from acute severe lumbar muscle tears or strains;

(5) LBP secondary to visceral organ pathologies, including nephrolithiasis, urinary tract infections, gynecological disorders, and malignant tumors;

(6) Concomitant severe systemic diseases such as uncontrolled hypertension or hematological disorders, or psychiatric disorders such as major depressive disorder or schizophrenia;

(7) Pregnant or lactating women;

(8) Presence of skin ulceration or infection at the treatment site;

(9) History of allergy to moxibustion or its components;

(10) Individuals who have received relevant treatments such as medications, physical therapy, or invasive procedures within the past 3 months, which could confound the study outcomes.

#### 2.3.3 Suspension criteria

(1) Patients who refuse to continue participation for personal reasons during the trial;

(2) Occurrence of serious adverse events (SAEs) directly attributable to the intervention, including skin burns, syncope, or other life-threatening complications;

(3) Participants demonstrating non-compliance with the study protocol, such as unscheduled treatment modifications or concurrent use of prohibited therapies.

### 2.4 Randomization

The study will employ a permuted block randomization method (with block size set to 4) stratified by research center for sequential allocation of participants. The randomization sequence will be generated by a statistician independent of the study team using SAS software (version 9.4; SAS Institute Inc., Cary, NC, USA). Research personnel will have no access to the randomization sequence. Randomization numbers will be sealed in sequentially numbered opaque envelopes and stored in a double-locked cabinet. Following completion of baseline assessments and submission of informed consent forms, envelopes will be opened in sequential order of enrollment to reveal group assignments. The allocation sequence will remain concealed such that the next participant’s assignment cannot be predicted in advance.

### 2.5 Blinding

Throughout the trial, the subjects will remain blinded to their group assignments. Specifically, each subject will be assigned to a separate treatment room to prevent participant-to-participant communication. Due to the unique characteristics of moxibustion therapy, it is difficult to implement blinding among the treating physicians. However, the data collectors, outcome assessors, and statisticians will be blinded to the details of treatment allocation and group assignments throughout the trial. Moreover, they will not be permitted to share any research-related information with each other. This approach will enhance the reliability of the study results. Unblinding will occur only in the event of serious adverse events or after study completion and final data analysis.

### 2.6 Interventions

#### 2.6.1 Moxibustion group

**FIGURE 2:**
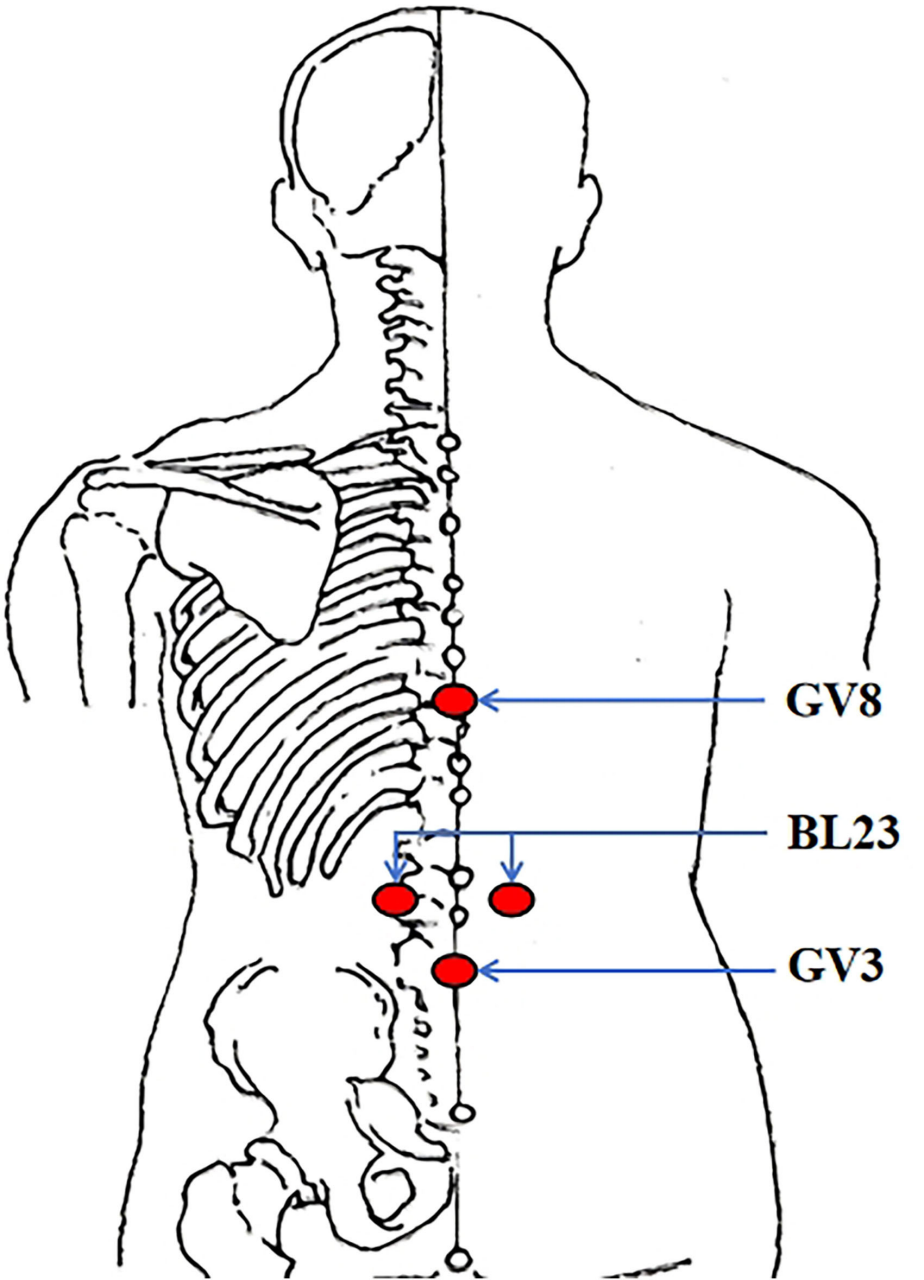
Location of acupoints. BL23 (Shenshu); under the spinous process of the second lumbar vertebra, 1.5 cun lateral to the posterior midline. GV3 (Yaoyangguan); on the posterior midline, under the spinous process of the fourth lumbar vertebra. GV8 (Jinsuo); on the posterior midline, under the spinous process of the ninth thoracic vertebra.

All practitioners in this trial must hold a qualified license in traditional Chinese medicine (TCM) and possess a minimum of 3 years of work experience. Moxibustion intervention will be carried out at the following acupoints: bilateral BL23 (Shenshu), GV3 (Yaoyangguan), and GV8 (Jinsuo) acupoints (Figure 2). After igniting one end of a moxa stick and securing it with a supporting device, the burning end will be positioned 3–5 cm above the designated acupoints, aiming to maintain the local skin temperature at 43 ± 1 °C. Participants will experience warmth without a burning sensation. Each moxibustion session will last 30 min, be administered three times per week, and continue for 8 weeks.

#### 2.6.2 Sham moxibustion group

Participants will undergo sham procedures at acupoints identical to those used for the true moxibustion group. After igniting and securing the moxa stick, the burning end will be placed 8–10 cm above the designated acupoints to maintain the local skin temperature at 37 ± 1 °C. To prevent thermal and smoke radiation from penetrating the skin, a heat-insulating metal membrane will be integrated into the base of the moxa stick holder. This modification has been proven in multiple previous studies to effectively block heat and smoke transmission (22–24). The moxa stick holder will have an identical appearance to that used in the moxibustion group. Participants will detect the aroma of burning moxa but will not experience thermal stimulation. The treatment duration and course will be consistent with those in the moxibustion group.

### 2.7 Outcome measures

The assessment of outcomes encompasses the following domains: low back pain intensity, somatic dysfunction, pain-related fear and avoidance behaviors, health status, and cerebral cortex activation characteristics. Assessments will be performed at baseline, mid-intervention (week 4), post-intervention (week 8), and follow-up (week 16). It is worth mentioning that participants will be required to rate the overall efficacy of the received intervention using the Global Perceived Effect (GPE), at week 8 (post-intervention). Additionally, functional near-infrared spectroscopy (fNIRS) will be employed by the researchers to assess cerebral cortex activation characteristics in each group at baseline and week 8 (end of intervention).

#### 2.7.1 Primary outcome

##### 2.7.1.1 Numeric Rating Scale (NRS)

This instrument serves as a standardized tool for assessing lumbar pain intensity, utilizing a numerical rating system spanning from 0 (indicating complete absence of pain) to 10 (representing the maximum tolerable pain threshold) (11). For patients with chronic LBP, a 2-point difference on the NRS was defined as the minimal clinically important difference (MCID) (25). The NRS score at week 8 will be regarded as the primary outcome measure.

#### 2.7.2 Secondary outcomes

##### 2.7.2.1 Oswestry Disability Index (ODI)

This questionnaire is designed to evaluate functional limitations caused by low back pain across ten domains (26). Each domain provides six response options scored from 0 to 5, where 0 indicates minimal or no disability and 5 indicates the most severe disability. Higher scores indicate greater disability. An MCID greater than 10 points is considered clinically significant for ODI (27).

##### 2.7.2.2 Fear-Avoidance Beliefs Questionnaire (FABQ)

The FABQ comprises two independent subscales and includes a total of 16 self-report items. The FABQ-Phys, which contains 5 items and has a total score range of 0 to 24, is intended to assess fear-avoidance beliefs related to general physical activities. The FABQ-Work, comprising 11 items with a total score range of 0 to 42, is employed to evaluate fear-avoidance beliefs associated with occupational activities. A higher score indicates a higher level of fear-avoidance beliefs (28). The MCID values for FABQ-Work and FABQ-Phys are 7 points and 4 points, respectively (29).

##### 2.7.2.3 36-Item Short Form Health Survey (SF-36)

This questionnaire consists of 36 questions that evaluate the impact of pain on patients’ daily health status across eight dimensions, and has been widely applied in medical research. The overall score spans a range from 0 up to 100, where lower numerical values are indicative of a diminished quality of life (30). Generally, an increase of 30% in the SF-36 score will be considered clinically significant (31).

##### 2.7.2.4 Global Perceived Effect (GPE)

This outcome measure employs a single-item self-assessment tool in which participants indicate their perceived changes in clinical status. The scoring range of this scale extends from −5 to +5. A score of + 5 is assigned when patients report complete resolution of pain symptoms following treatment, whereas a score of 0 indicates no perceived change in pain symptoms, and a score of −5 reflects significant exacerbation of pain symptoms after treatment (32). The MCID has been defined as 1.7 points for chronic LBP (33).

##### 2.7.2.5 fNIRS

Pain is closely associated with the functional connectivity of relevant cerebral cortical regions. fNIRS is an emerging optical imaging technique that reflects cortical activation patterns associated with neuronal activity by detecting concentration changes in oxygenated hemoglobin (HbO_2_) within localized cerebral regions (34). fNIRS examinations will be conducted in a quiet room with subdued lighting. Data acquisition will be performed using a 48-channel near-infrared optical imaging system (NirScan-6000A, HuiChuang Medical Equipment Co., Ltd., Danyang, China). This system operates at two wavelengths (730 nm, and 850 nm) with a sampling rate of 11 Hz. It comprises 15 light source optodes and 16 photodetector probes, with an optode-to-detector distance of 3 cm. Light sources and detectors will be symmetrically distributed over the bilateral dorsolateral prefrontal cortex (DLPFC), primary motor cortex (M1), primary somatosensory cortex (S1), premotor cortex (PMC), and supplementary motor area (SMA) in accordance with the international 10-20 standard electrode placement system. Acquired data will be analyzed using the NirSpark software.

### 2.8 Safety evaluation and adverse events (AEs)

Continuous safety monitoring of participants will be required during each intervention session throughout the trial. All AEs that occur during the intervention or follow-up periods will be systematically documented in the case report forms (CRF), along with a careful assessment of their potential association with moxibustion therapy. Potential risks associated with moxibustion include skin burns, blistering, pruritus, dizziness, and other post-moxibustion discomforts. In the event of an AE, regardless of its perceived relationship to the study, qualified medical professionals will be required to provide immediate symptomatic treatment and appropriate interventions. Researchers will complete detailed documentation in the CRF regarding the AE’s onset time, specific manifestations, severity grading, therapeutic measures implemented, and clinical outcomes. Safety monitoring will continue until resolution or stabilization of the participant’s condition. All AEs will be reported to the trial’s principal investigator (PI) and the institutional ethics committee for review, followed by subsequent evaluation of the participant’s eligibility for continued trial participation. Compensation procedures will be activated for study-related serious adverse events (SAEs) in accordance with regulatory requirements and institutional policies.

### 2.9 Data management and quality control

The data collected by professional physicians will be stored in a dedicated electronic database, and the participants’ identifying information will be concealed using numerical codes. Each participating research center will be responsible for monitoring data quality. To maintain procedural consistency across study centers, quarterly investigator meetings are planned to address emerging trial-related issues and facilitate ongoing communication with all research personnel. To enhance participant compliance, researchers will provide timely reminders via telephone for scheduled visits and assessments. For subjects who withdraw from the clinical trial or follow-up, detailed documentation of reasons and outcomes will be systematically recorded.

### 2.10 Sample size calculation

The sample size calculation was performed using G*Power software (version 3.1.9.7; University of Düsseldorf, Germany). The calculation was based on a prior clinical trial evaluating acupuncture for CNLBP, which also used the NRS as the primary outcome measure (35). According to the reported data from that study, the post-treatment mean ± standard deviation (SD) was 2.96 ± 3.44 for the treatment group (*n* = 29) and 2.00 ± 2.79 for the control group (*n* = 28), yielding an effect size of 0.3 (Cohen’s d), suggesting a small intervention effect. Considering subjective feedback from CNLBP patients who had previously received moxibustion therapy regarding its perceived efficacy, we estimated a potential effect size of 0.5 (moderate effect) for moxibustion treatment. With α set at 0.05, power (1-β) at 0.80, and these parameters entered into G*Power, the calculation indicated a required sample size of 64 participants per group. Accounting for an expected 15% dropout rate during the trial, the total sample size was adjusted to 150 participants (75 per group).

### 2.11 Statistical analysis

To minimize potential biases arising from manual intervention, the statistical analyses for this study will be conducted independently by two blinded statisticians using SPSS (version 25), both of whom will be unaware of the group assignments and treatment details. Data analysis will adhere to the intention-to-treat principle. Participants who complete the baseline assessment of the primary outcome and receive at least one session of moxibustion or sham moxibustion treatment will be included in the ITT analysis. Continuous variables will be presented as mean ± standard deviation (SD) or median (P25-P75), with statistical comparisons performed using Student’s *t*-test or non-parametric Mann-Whitney U test as appropriate. Categorical variables will be described as frequencies and percentages, with between-group differences analyzed via χ^2^ test or Fisher’s exact test. It should be noted that all baseline comparisons serve solely to verify inter-group balance after randomization, and their results will not be used to adjust the primary analysis model. For repeatedly measured outcome variables, a random-intercept mixed-effects model (MMRM) will be employed. Specifically, the model will include observed values at each scheduled assessment timepoint (weeks 4, 8, and 16) as dependent variables, with baseline values incorporated as fixed-effect covariates. Treatment group (moxibustion vs. sham moxibustion), timepoint (weeks 4, 8, and 16), and their interaction will be modeled as fixed-effect categorical variables. Additionally, individual subject intercepts will be included as random effects. Between-group comparisons at each timepoint will be estimated through least squares mean differences derived from treatment-by-time interaction terms, with corresponding *P*-values and 95% confidence intervals (CI) reported. All statistical tests will be two-sided, and a *P*-value < 0.05 will be considered statistically significant.

## 3 Discussion

Chronic non-specific low back pain has been emerging as a leading cause of population-based disability worldwide for decades (36). Current guidelines recommend physical therapy as a first-line treatment for CNLBP. However, patients do not always derive sufficient benefits from physical therapy alone. Coupled with the potential adverse effects and addiction risks associated with pharmacological interventions, there is a growing demand for safer and more effective therapeutic options for CNLBP (37). Moxibustion, a key component of TCM, has contributed to the health of the Chinese population for thousands of years. Clinical observations suggest that moxibustion can alleviate pain and somatic dysfunction in patients with CNLBP to some extent, positioning it as a promising complementary therapeutic approach. Nevertheless, rigorously conducted clinical trials and robust evidence supporting moxibustion for CNLBP remain scarce, and the precise mechanism of action underlying moxibustion remains incompletely elucidated. Therefore, we conducted this meticulously designed multicenter, single-blind randomized controlled trial to validate the efficacy of moxibustion in patients with CNLBP.

According to TCM theory, acupoints exhibit local therapeutic effects, meaning each acupoint located at a specific anatomical position can treat diseases in its vicinity and adjacent tissues and organs. These acupoints, termed “local acupoints”, are commonly used for managing musculoskeletal disorders and somatic pain conditions (38). In this trial, we selected Shenshu (BL23), Yaoyangguan (GV3), and Jinsuo (GV8), all situated in the lumbosacral region and frequently utilized in clinical acupuncture practice for chronic LBP (38, 39). Unlike sole reliance on herbal pharmacotherapy, the therapeutic effects of moxibustion arise from the dynamic interaction between local physical stimulation and the body’s intrinsic regulatory systems. This mechanism embodies the distinctive therapeutic philosophy underlying TCM approaches to disease management. Due to the inherent methodological challenges posed by the well-documented placebo effect in TCM complementary therapies, the implementation of a credible sham moxibustion control condition became an essential component of the study design. It is well-established that temperature is a pivotal factor influencing the therapeutic outcomes of moxibustion treatment. This is because when there is effective heat insulation, the clinical benefits derived from moxibustion are markedly diminished. In the current study, the sham moxibustion intervention was applied at a distance of 8–10 cm with temperature maintained at 37 ± 1 °C. This protocol minimized thermal stimulation while allowing participants to perceive the characteristic odor of burning moxa, thereby achieving effective blinding (40, 41).

Pain is the predominant symptom of CNLBP. Consequently, the NRS was adopted to evaluate whether the intervention could alleviate patients’ pain intensity. For individuals with CNLBP, psychological factors serve as critical predictors of long-term disability, with fear of pain often contributing more to functional limitations than the pain itself (42, 43). Fear-avoidance beliefs may drive patients to adopt protective behaviors, thereby precipitating and perpetuating disability while hindering the restoration of normal functional capacity (44, 45). Therefore, the FABQ and the ODI were employed to assess whether the intervention could ameliorate pain-related fear and associated physical dysfunction. Given that persistent pain severely compromises patients’ physical and mental wellbeing, the SF-36 was additionally utilized to comprehensively evaluate whether the intervention could enhance patients’ quality of daily life.

The ambiguous action mechanism of moxibustion therapy stands as one of the crucial factors restricting its global promotion and application. As is widely acknowledged, the experience of pain extends beyond localized pathological alterations at the site of injury to encompass the neural processing within cortical regions mediating pain perception. Thus, it is highly worthwhile to investigate whether the mechanism by which moxibustion alleviates pain is linked to its ability to regulate the functional activity of pain-related areas in the cerebral cortex. Previous studies have demonstrated that CNLBP induces structural and functional alterations in brain regions associated with pain modulation and postural control, with impaired postural control potentially contributing to the persistence and recurrence of pain in CNLBP patients (46, 47). In this study, we selected bilateral DLPFC, M1, S1, SMA, and PMC as regions of interest, as these cortical areas play critical roles in human pain modulation and postural control (48, 49). fNIRS will be employed to compare activation patterns of these cortical regions in participants before and after intervention, which will help elucidate the potential neurophysiological mechanisms underlying moxibustion therapy for CNLBP.

However, limitations exist in this trial. Firstly, owing to the unique characteristic of moxibustion therapy, achieving true blinding for clinical practitioners remains challenging, which may introduce potential performance bias. Independent assessment of outcome measures will help resolve this issue. Secondly, participants’ high expectations regarding the efficacy of moxibustion may introduce bias into the outcomes.

This trial may have some significant implications. To begin with, this study aims to comprehensively assess the diverse impacts that moxibustion exerts on pain severity, physical functional impairment, pain-related fear, and the overall quality of life among patients suffering from CNLBP. If the results support our hypotheses, moxibustion therapy could be widely implemented in future clinical practice. Secondly, this study will investigate whether therapeutic efficacy of moxibustion for CNLBP varies with temperature parameters, which will provide data-driven evidence to optimize treatment protocols. Lastly, fNIRS will be employed in this study to investigate cortical activation patterns in patients with CNLBP, thereby elucidating the neurobiological mechanisms underlying moxibustion’s therapeutic effects from a modern scientific perspective. In summary, this study may provide a complementary therapeutic strategy for CNLBP management and may promote further research on moxibustion treatment in the future.
